# A type 6 secretion system (T6SS) encoded gene within *Salmonella enterica* serovar Enteritidis contributes to virulence

**DOI:** 10.1080/21505594.2017.1421829

**Published:** 2018-02-27

**Authors:** Bryan Troxell

**Affiliations:** Alcami Corporation, Biotechnology department, Durham, North Carolina, USA

**Keywords:** *Salmonella*, invasion, motility, protein secretion, gene regulation

## Abstract

Bacteria interact with their host through protein secretion systems and surface structures. Pathogenic bacteria encode protein secretion systems that promote the invasion of the host's tissue, the evasion of the host's immune response, the thwarting microbial competitors, and ultimately survival within the host. For motile bacteria, the presence of extracellular flagella provides the host with a structural motif used for activation of the immune system. Within this issue of *Virulence*, the article “Identification of a novel gene in ROD9 island of *Salmonella* Enteritidis involved in the alteration of virulence-associated protein expression” describes the contribution of a gene, *SEN1005*, toward host-pathogen interaction. The authors demonstrate the contribution of *SEN1005* to cell culture bioassays and infection in a mouse model of colitis. In each tested scenario, deletion of *SEN1005* results in a phenotypic defect that was complemented by providing the *SEN1005* gene *in trans*. SEN1005 contributes to the expression of known virulence factors within SPI-1, flagellar and chemotaxis genes, and heat shock/chaperone genes. Although much work is needed to fully elucidate the function of SEN1005, this work contributes toward our understanding of the genetic factors used by *Salmonella* to cause foodborne illnesses.

*Salmonella* encodes numerous pathogenicity islands (SPIs). SPI-1 and SPI-2 are the most studied and contribute to virulence via invasion of the epithelial cell layer and survival within systemic tissues, respectively [1–6]. SPI-1 is encoded in all *Salmonella* genomes identified thus far, whereas SPI-2 is absent from the genome of *Salmonella bongori* [7]. In addition, there are other SPIs that are not present in all serovars [8]. The region of differences (ROD) 9, also referred to as SPI-19, was previously identified using a bioinformatic screen for type 6 secretion systems (T6SS) within *Salmonella* serovars [9]. T6SS are a protein secretion system that are directly involved in microbial competition [10]. ROD9 is intact in serovars Dublin, Weltevreden, Agona, and Gallinarum. Interestingly, Enteritidis is missing an ∼24 kb segment [9, suggesting ROD9 may not function as a T6SS within Enteritidis. Although the contribution of ROD9 to microbial competition is unknown, earlier work demonstrated that the Enteritidis strain (P125109) used by Das *et al* [11] colonized the streptomycin treated murine tissues to a higher level than that of Typhimurium [12,13]. Whether this is solely due to ROD9 or to a collection of factors is currently unknown.

The truncated ROD9 in Enterititidis potentially encodes 16 open reading frames (ORFs). The authors tested several of these genes for phenotypes in two bioassays that measured the adhesion and invasion of an epithelial cell line. Of the six mutant strains examined, only *SEN1008* and *SEN1005* demonstrated a significant defect; Δ*SEN1008* was defective in adhesion whereas Δ*SEN1005* was defective in invasion. Both were also defective in uptake by the murine macrophage cell line RAW264.7. The authors focused on *SEN1005* for the remainder of their studies. Motility is associated with invasion of host cells and uptake by phagocytic cells [14–21]; therefore, the authors tested the mobility of the Δ*SEN1005* strain. Under the tested conditions, the mutant displays reduced motility and expression of the flagellin structural gene, *fliC*. Moreover, SPI-1 genes exhibit reduced expression, most notably the master regulator of SPI-1, *hilD*. HilD also activates *flhDC*, whose gene products are required for activation of the flagellar class 2 promoters [22,23]. Flagellin is a pathogen-associated molecular patterns (PAMPs) that is recognized by the toll-like receptor 5 (TLR5). Recognition of flagellin by TLR5 signals activation of the nuclear factor kappa-light-chain-enhancer of activated B cells (NF-κB) and tumor necrosis factor alpha (TNFα) [24]. The reduced expression of *fliC* in Δ*SEN1005* may account for a majority of the observed phenotypes described by Das *et al*, such as the reduced expression of interleukin 1 (IL-1), IL-8, TNFα, interferon gamma (INFγ), and lowered nitric oxide (NO) production by host cells.

How does *SEN1005* contribute to virulence in Enteritidis? The reduced expression of SPI-1, flagella, and chemotaxis genes may be contributors to the observed *in vivo* phenotypes of Δ*SEN1005*. Noticeably, Δ*SEN1005* also exhibited the increased expression of genes whose products are involved in the heat shock response. The heat shock response includes the induced expression and also activation of numerous chaperones and proteases [25]. *ΔSEN1005* exhibits increased expression of the major heat shock/chaperone genes, *dnaK* and *groES*, which are directly controlled by the alternative sigma factor σ^32^ (RpoH). The cellular content of RpoH is kept low under basal conditions, but exposure to increased temperatures, low pH, oxidative stress, and membrane disruption causes the increased abundance of active RpoH [25]. This regulatory pathway is known to reduce expression of SPI-1 in Typhimurium [26]. An independent pathway that senses membrane disruption, the two-component system (TCS) CpxAR system, may also be involved. CpxA is histidine kinase that activates CpxR; however, CpxA also acts as a phosphatase that maintains inactive CpxR under appropriate conditions [27]. A speculation from the current data would be that deletion of *SEN1005* results in membrane disruption that activates either RpoH or CpxR. Both of these pathways result in reduced *hilD* expression or HilD activation [26,28]. Since the function of SEN1005 is currently unknown the contribution of this gene to Enteritidis virulence is open to numerous possibilities. One caveat to these comparisons is that a majority of these studies were conducted with serovar Typhimurium. Whether CpxAR exerts the same effect in Enteritidis is unknown, but earlier work suggests that the heat shock response does represses SPI-1 in both Typhimurium and Enteritidis [29].

Non-typhoidal *Salmonella* infections are a global health problem. The ability of these pathogens to colonize and persistent within different animal hosts contribute to the difficulty in managing and implementing preventative measures. Despite the near identical genetic composition among the > 2,500 serovars, a difference in host specificity and *in vivo* fitness is apparent. The human specific serovars, which can cause lethal enteric fever have genetic factors important for low-level inflammation and systemic colonization of their hosts, but lack factors important for colonization of other warm blooded animals. Conversely, the non-typhoidal serovars are capable of causing self-limiting, but inflammatory phenotypes in an array of hosts. The absence, presence, and partial loss of ROD9 in serovar genomes suggest that altered selective pressures have been exerted on this island. Whether *SEN1005* or other genes within ROD9 (SPI-19) contribute to host specificity requires future work.
